# Worldwide Prevalence of Occupational Exposure to Needle Stick Injury among Healthcare Workers: A Systematic Review and Meta-Analysis

**DOI:** 10.1155/2021/9019534

**Published:** 2021-01-29

**Authors:** Dechasa Adare Mengistu, Sina Temesgen Tolera, Yohannes Mulugeta Demmu

**Affiliations:** Department of Environmental Health, College of Health and Medical Science, Haramaya University, Harar, Ethiopia

## Abstract

**Background:**

Healthcare workers are at high risk of occupational exposure to needle stick injury worldwide. Occupational exposure to needle stick injury represents the most common sources of infection such as hepatitis B virus, hepatitis C virus, and human immunodeficiency virus. Thus, this review aimed to determine the career time and previous one-year global pooled prevalence of occupational exposure to needle stick injury among healthcare workers.

**Methods:**

The review considered articles written in English language and published from 2012 to 2020. The articles were searched using nine electronic databases (PubMed, Google Scholar, CINAHL, MEDLINE, Cochrane library, Web of Science, SCOPUS, MedNar, and ScienceDirect) using a combination of Boolean logic operators (AND, OR, and NOT), Medical Subject Headings, and keywords. Quality assessment was performed to determine the relevance of the articles using Joanna Briggs Institute critical appraisal tools. Several steps of assessment and evaluation were taken to select and analyze the relevant articles.

**Results:**

The worldwide pooled prevalence of needle stick injuries among healthcare workers during career time and previous one year was 56.2% (95% CI: 47.1, 64.9) and 32.4% (95% CI: 22.0, 44.8), respectively. The career time pooled prevalence of needle stick injuries based on the socioeconomic development and study area was 54.8% and 55.1%, respectively, and one-year pooled prevalence of needle stick injury was 26.0% and 20.9%.

**Conclusion:**

The review found a high prevalence of occupational exposure to needle stick injury among healthcare workers and suggests the need to improve occupational health and safety services in the healthcare systems.

## 1. Introduction

Needle stick injuries (NSIs) are among the most common occupational hazards among healthcare workers (HCWs) worldwide that need to be addressed and represent the most common sources of infection [1]. Infectious complications related to occupational exposure to NSI can result in serious health problems ranging from mild to extreme anxiety [2].

Today, at least 20 different pathogens are transmitted by NSIs such as hepatitis B virus (HBV), hepatitis C virus (HCV), and human immunodeficiency virus (HIV) [3, 4]. Annually, hundreds of thousands of HCWs are at high risk of work-related infections such as HBV, HCV, and HIV as a result of exposure to contaminated needle sticks and sharp injuries [5, 6]. Furthermore, the risk of infections from NSIs ranges from 0.2 to 0.5% for HIV, 3–10% for HCV, and 40% for HBV [7].

According to Centers for Disease Control and Prevention (CDC) and European Agency for Safety and Health at Work, more than 385,000 and one million NSIs cases are reported annually among HCWs working in hospitals in the United States and Europe, respectively [8, 9]. Worldwide, about three million of HCWs were exposed to blood pathogens through percutaneous, of which two million were exposed to HBV, 0.9 million exposed to HCV, and 170,000 exposed to HIV of which more than 90% occurred in developing countries [10–12].

World Health Organization (WHO) estimated that NSIs cause HCV that account 16,000, HBV that account 66,000, and HIV that account 1,000 annually among HCWs [13]. Furthermore, percutaneous exposure accounts for approximately 37.0% of HBV, 39.0% of HCV, and 4.4% of HIV cases among HCWs [14].

Determining the worldwide prevalence of needle stick injury among HCWs is necessary, particularly in reducing NSI, creating safer working conditions and cultures, reducing costs, and provision of higher quality services [15–17].

Prior to this study, many studies have reported the prevalence of NSI among healthcare workers at the country, regional, or institutional level [18–22]. Also, there are a few studies that provide worldwide evidence of the prevalence of occupational exposure to NSIs among HCWs and reported one-year prevalence alone [1]. Thus, this study aimed to determine and provide the worldwide pooled prevalence of needle stick injury (both one year and career time prevalence) among healthcare workers that are very important for understanding the problems and designing prevention program including occupational health and safety practices and standard precautions.

## 2. Methods

### 2.1. Eligibility Criteria

The articles that met the following inclusion criteria were included in the systematic review and meta-analysis:

 
*(i). Study Design.* Cross-sectional studies
 
*(ii). Outcome.* Studies that provide quantitated outcomes (magnitude, frequency, or prevalence of NSI)
 
*(iii). Study Area.* Studies conducted in developed and/or developing countries
 
*(iv). Language.* Full-text articles published in the English language
 
*(v). Population.* Healthcare workers and medical students regardless of their occupation
 
*(vi). Publication Issue.* Articles published in peer-reviewed journals from 2012 to 2020

On the contrary, the studies reported period prevalence (such as 3 or/and 6 months) of NSIs, case reports, case series, qualitative studies, review articles, surveillance data/reports, conference abstracts, personal opinions, non-healthcare workers study participants, studies that utilized less than 120 participants, articles written in non-English language, high risk of bias articles, study not available in full texts, and studies published before 2012 were excluded from the study.

### 2.2. Information Sources and Search Strategy

The articles were searched using nine electronic databases (PubMed, Google Scholar, CINAHL, MEDLINE, Cochrane library, Web of Science, SCOPUS, MedNar, and ScienceDirect) using a combination of Boolean logic operators (AND, OR, and NOT), Medical Subject Headings, and keywords such as health professionals, healthcare workers, healthcare system, developing country, developed country, needle stick injury, and occupational exposure.

The articles were searched using a combination of Boolean logic operators (AND, OR, and NOT), Medical Subject Headings (MeSH), and keywords. The following is a search term used in the initial searching (((“prevalence”[MeSH Terms] OR “prevalence”[All Fields]) AND ((“occupational”[MeSH Terms] OR “occupational”[All Fields], OR “work place”[All Fields] OR “workplace”[MeSH])) AND ((“needle stick injury”[MeSH Terms] OR (“needle stick”[All Fields] AND “injury”[All Fields]) OR “needle stick injury” [All Fields])) AND ((“healthcare workers” [MeSH Terms] OR “healthcare”[All Fields] AND “workers”[All Fields]) OR “healthcare workers”[All Fields])) OR ((“health professional”[MeSH Terms] OR (“health”[All Fields] AND “professional”[All Fields]) OR “health professional”[All Fields])) OR ((“health provider”[MeSH Terms] OR (“health”[All Fields] AND “provider”[All Fields]) OR “health provider”[All Fields])) AND ((“developing country”[MeSH Terms] OR (“developing”[All Fields] AND “countries”[All Fields]) OR “developing countries”[All Fields])) OR ((“developed countries”[MeSH Terms] OR (“developed”[All Fields] AND “countries”[All Fields]) OR “developed countries”[All Fields])).

Then, all identified keywords and an index term were checked across the included nine electronic databases. Finally, searching the reference list of all identified articles for further articles was conducted.

### 2.3. Study Selection

Duplicated articles were removed using the ENDNOTE software version X5 (Thomson Reuters, USA). The authors (DA. Mengistu, ST. Tolera, and YM. Demmu) screened the titles and abstracts of the identified articles by applying the inclusion and exclusion criteria. Finally, the review included only articles conducted to determine the prevalence of NSIs among healthcare workers in healthcare systems of developing or developed countries.

### 2.4. Quality Assessment

Full-text articles, available in English language, with clear objectives and methodology, and studies including needle stick injury as a dependent variable and providing quantitative outcomes were selected. These articles were then evaluated to confirm their relevance to the study and to confirm the quality of the work.

Furthermore, selected articles were subjected to a rigorous, independent appraisal using standardized critical appraisal tools (JBI Critical Appraisal tools) [23] to determine the quality and relevance of the articles. Then, the score was taken across all studies and graded as high (85% and above score), moderate (60–85% score), and low (<60% score) quality. Disagreement made on what is to be extracted was solved by discussion. The PRISMA guidelines protocol [24] was used to conduct the review.

### 2.5. Data Extraction

The authors (DA. Mengistu, ST. Tolera, and YM. Demmu) independently extracted data from the included articles. A predefined Microsoft Excel 2016 format was used to extract information from selected studies under the following headings: author, publication year, country of study, study design, and primary outcomes such as prevalence or magnitude of exposure to NSI and possible confounding factors considered. In general, all required data were extracted from the eligible articles.

### 2.6. Data Analysis and Statistical Procedures

The prevalence of NSI was categorized into career time and 12-month prevalence. For those studies reporting the frequency of NSI without calculating the prevalence, the prevalence was calculated by dividing the frequency of exposed to the total sample size or multiplying the ratio of exposed to sample size.

The pooled prevalence of both NSIS was done using Comprehensive Meta-Analysis (CMA) version 3.0 statistical software. The random-effect model and forest plot were used to estimate the pooled prevalence of needle stick injury among healthcare workers with 95% confidence intervals (95% CI). The possibility of publication bias was assessed by visual funnel plots, and a *p* value <0.05 was considered as the evidence for publication bias. Furthermore, subgroup analysis was conducted based on the countries where the articles were conducted, and socioeconomic development, to minimize the random variations (heterogeneity) between the point estimates of the included articles.

### 2.7. Heterogeneity

In this study, Cochran's *Q* test (*Q*) and (I Squared test) I^2^ statistics were used to evaluate the heterogeneity of the included articles. Furthermore, the differences among included articles were evaluated using graphical and statistical tests. Then, the characteristics of the articles were described using texts, tables, and forest plots. Forest plot was used to evaluate the pooled prevalence of both NSIs. Subgroup analysis was done based on study area and socioeconomic development.

## 3. Results

### 3.1. Study Selection

A total of 3,018 studies published from 2012 to 2020 were identified. Then, 976 duplicate articles were excluded, while 1,781 articles were excluded based on the exclusion criteria. Furthermore, 161 full-text studies were further assessed to determine their eligibility, of which 143 studies were excluded as they failed to report the prevalence of needle stick injuries, due to their unclear objectives, unclear methods, small sample size, and non-healthcare worker participants.

**Figure 1:**
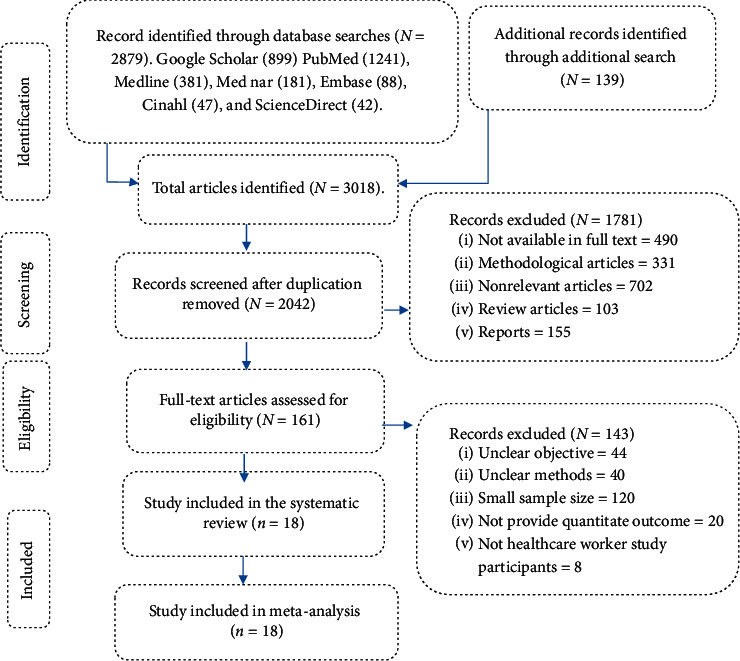
PRISMA flow diagram shows the selection process of included articles for a systematic review and meta-analysis.

Finally, 18 articles that met the inclusion criteria were included in the review (Figure 1).

### 3.2. Study Characteristics

A total of 10,233 healthcare workers were included in 18 studies, of which were conducted in 14 countries [16, 25–41]: three articles [29, 33, 41] in Iran, two in Ethiopia [27, 36], two in India [28, 39], and one (5.55%) in other countries such as Nigeria [30], USA [25], China [31], Serbia [26], Saudi Arabia [32], Bangalore [34], Thailand [16], Australia [35], Bosnia and Herzegovina [37], Tanzania [38], and Switzerland [40].

The included studies had a sample size ranging from 120 [30] to 2691 [40] HCWs. Based on JBI Critical Appraisal tool, 15 (83.3%) of the included articles had a low risk of bias, while the remaining three had a medium risk of bias. The career time and previous one-year prevalence of NSIs among HCWs was in the range from 29.8% [32] to 100% [33] and from 9.7% [40] to 81.7% [33], respectively.

Among the included articles, 6 articles [26–30, 33] reported both prevalence of NSIs in career time and previous one year, while 6 [25, 31, 32, 36, 37, 41] and 6 [16, 34, 35, 38–40] of articles reported career time alone and previous one-year prevalence of NSIs alone, respectively. Most ( 5 (83.3%)) of the included articles [16, 26–34, 36–39, 41] were conducted in the developing countries, while the rest of the studies [25, 35, 40] were in a developed country (Table 1).

### 3.3. Prevalence of Needle Stick Injury

#### 3.3.1. Career Time Prevalence of Needle Stick Injury

**Figure 2:**
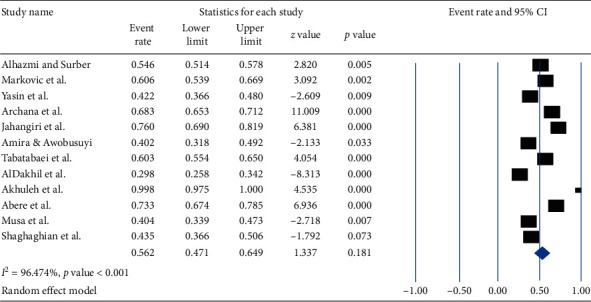
Forest plot shows the pooled prevalence of career time occupational exposure to needle stick injury among healthcare workers.

The career time prevalence of occupational exposure to needle stick injury among healthcare workers was 56.2% (95% CI of 47.1 to 64.9), with I^2^ = 96.474% and a *p* value < 0.001 (Figure 2).

**Figure 3:**
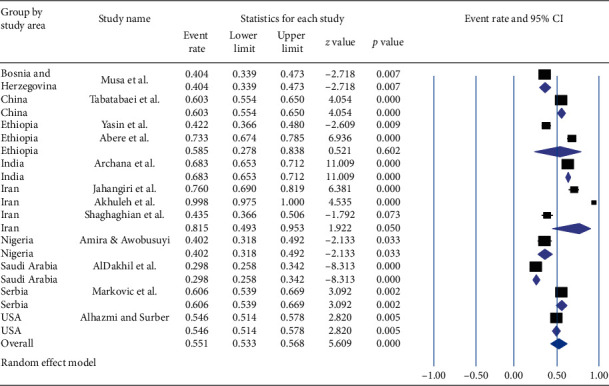
Forest plot shows the subgroup analysis of pooled prevalence of career time occupational exposure to needle stick injury based on the study area.

Based on a subgroup analysis by country where the studies are conducted, the lowest prevalence (29.8% (95% CI: 25.8, 34.2%) with a value of <0.001) of career time exposure to NSI among HCWs was observed in Saudi Arabia, whereas the highest prevalence (81.5%, (95% CI: 49.3–95.3%) with a *p* value of 0.05) was in Iran. The overall pooled prevalence of career time occupational exposure to NSI was 55.1% (95% CI: 53.3–56.8%) with a value of *p* < 0.001 (Figure 3).

**Figure 4:**
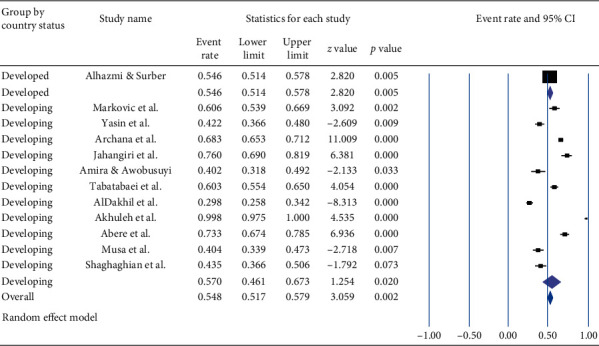
Forest plot shows the subgroup analysis of pooled prevalence of career time occupational exposure to needle stick injury based on socioeconomic development.

Based on socioeconomic development, the lowest pooled prevalence (54.6% (95% CI: 51.4, 57.8%) with a *p* value of 0.005) of career time occupational exposure to NSI was in developed countries while the highest pooled prevalence (57.0%, (95% CI: 46.1–67.3%) with a *p* value of 0.02) was in developing countries. The overall pooled prevalence of career time occupational exposure to NSI among HCWs was 54.8% (95% CI: 51.7–57.9%) with a *p* value of 0.002 (Figure 4).

#### 3.3.2. Previous One-Year Prevalence of Needle Stick Injury

**Figure 5:**
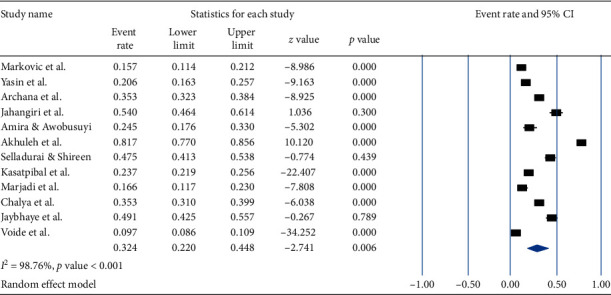
Forest plot shows the pooled prevalence of occupational exposure to needle stick injury in the previous one year.

The pooled prevalence of occupational exposure to needle stick injury among HCWs in the previous 12 months was 32.4% (95% CI: 22.0, 44.8 and a *p* value = 0.006) with *I*^2^ = 98.76% and a *p* value of <0.001 (Figure 5).

**Figure 6:**
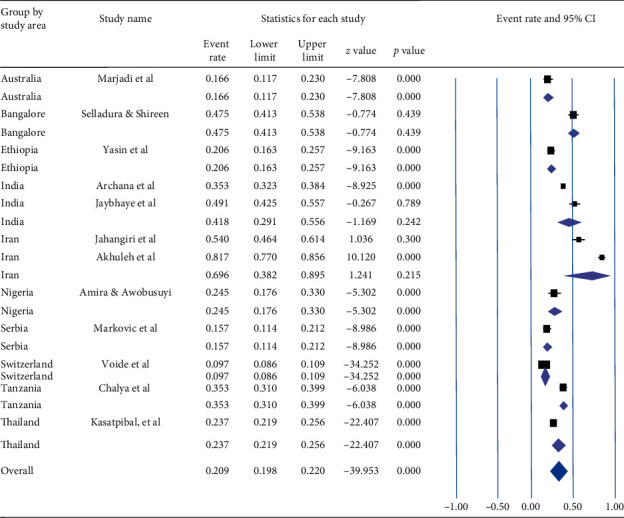
Forest plot shows the subgroup analysis of pooled prevalence of needle stick injury during the previous one year based on the study area.

Based on a subgroup analysis by countries where the studies are conducted, the lowest prevalence [9.7% (95% CI: 8.6%–10.9%)] of NSI in the previous one year was observed in Switzerland, whereas the highest prevalence [69.6%, (95% CI: 38.2–89.5%)] of NSI was observed among the studies conducted in Iran. The overall pooled prevalence during the previous one year was 20.9% (95% CI: 19.8–22.0%) with a value of *p* < 0.001 (Figure 6).

**Figure 7:**
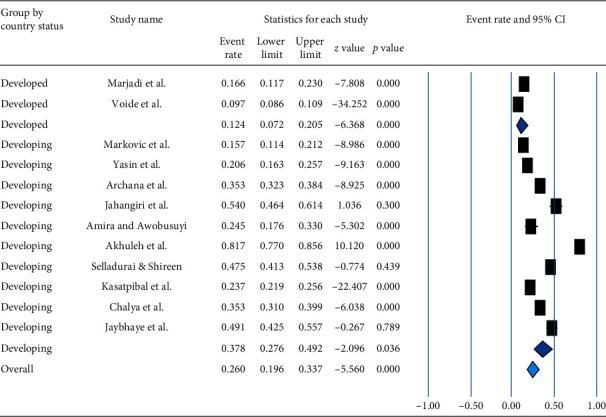
Forest plot shows the subgroup analysis of pooled prevalence of needle stick injury in the previous one year based on socioeconomic development.

Based on socioeconomic development, the pooled prevalence of NSI among HCWs in the previous one year was 12.4% (95% CI: 7.2%–20.5%, a *p* value <0.0001) and 37.8% (95% CI: 27.6–49.2%, a *p* value of 0.036) in developed and developing countries, respectively. The overall pooled prevalence of NSI was 26.0% (95% CI: 19.6–33.7%) with a *p* value of <0.001 (Figure 7).

## 4. Discussion

Occupational exposure to NSIs is a major source for the transmission of blood-borne pathogens such as HBV, HCV, and HIV. However, the current review found the pooled prevalence of needle stick injury among HCWs during career time and previous one year accounted 56.2% and 32.4%, respectively. Also, we found a lower pooled prevalence of the previous one-year needle stick injury than the prevalence of NSI injury reported by Bouya et al., 2020 (44.5%; 95% CI 33.7, 53.2) [1]. Auta et al., 2018 [42], also reported the one-year global pooled prevalence of percutaneous injuries that accounted 36.4% (95% CI: 32.9, 40.0) that was higher than our estimates.

The pooled prevalence of needle stick injury among HCWs during their career time and in the previous one year varied based on publication year, socioeconomic development, and study area (country). This finding may be related to the variation in the application of standard procedures, occupational health and safety systems, availability and implementation of policies, poor NSI management, and unsafe working environments. The health problems related to occupational exposure to NSI such as HBV, HCV, and HIV infections were higher in developing countries [10, 13, 42, 43]. Our review also found the pooled prevalence of NSIs during career time and previous one year among HCWs in developing countries was higher than in developed countries.

Overall, the review reported a high prevalence of NSIs among HCWs; thus occupational health and safety are crucial to reduce the risk of occupational exposure to NSI and the transmission of infectious diseases. Applying at least the following principles such as (1) establishing and implementing policies on NSIs management, (2) creating an appropriate safety and organizational culture, (3) applying standard precautions, (4) regular training on infection prevention and standard precautions, (5) regularly monitoring the proper implementation of guidelines, and (6) developing long-term NSIs reporting systems that play a great role in reducing NSIs and preventing infectious disease [1, 10, 42–45].

Our review included studies from only fourteen countries. Most of these studies were conducted in developing countries, which limits the interpretation of results. Furthermore, the included articles were cross-sectional studies and the methodological limitations of such studies need to be considered when interpreting their results. Also, data from most studies were collected based on a self-reported manner and this can affect the prevalence of needle stick injury due to reporting of exposure.

## 5. Conclusion

The review found a high prevalence of occupational exposure to needle stick injury among HCWs and suggests the need to improve occupational health and safety services in healthcare system globally. Thus, applying standard precautions, regularly training on infection prevention, and regularly monitoring the proper implementation of guidelines play a great role in reducing NSIs and preventing infectious diseases.

## Figures and Tables

**Table 1:** Overall characteristics of included articles in the systematic review and meta-analysis.

Authors (publication year)	*N*	NSI prevalence		Study design	Location	Socioeconomic development	Risk of bias	Reference
		Life time	One year					
Alhazmi and Surber, 2018	926	54.64	NA	Cross-sectional	USA	Developed	Low	[25]
Marković et al., 2013	216	60.6	15.7	Cross-sectional	Serbia	Developing	Low	[26]
Yasin et al., 2019	282	42.2	20.6	Cross-sectional	Ethiopia	Developing	Low	[27]
Archana et al., 2018	950	68.3	35.3	Cross-sectional	India	Developing	Low	[28]
Jahangiri et al., 2015	168	76.0	54.0	Cross-sectional	Iran	Developing	Low	[29]
Amira and Awobusuyi, 2014	120	40.2	24.5	Cross-sectional	Nigeria	Developing	Low	[30]
Tabatabaei et al., 2016	393	60.3	NA	Cross-sectional	China	Developing	Low	[31]
AlDakhil et al., 2019	450	29.8	NA	Cross-sectional	Saudi Arabia	Developing	Low	[32]
Akhuleh et al., 2019	306	100	81.7	Cross-sectional	Iran	Developing	Low	[33]
Selladurai, and Shireen, 2019	240	NA	47.5	Cross-sectional	Bangalore	Developing	Low	[34]
Kasatpibal et al., 2016	2031	NA	23.7	Cross-sectional	Thailand	Developing	Low	[16]
Marjadi et al., 2017	169	NA	16.6	Cross-sectional	Australia	Developed	Low	[35]
Abere et al., 2020	241	73.3	NA	Cross-sectional	Ethiopia	Developing	Low	[36]
Musa et al., 2014	203	40.4	NA	Cross-sectional	Bosnia and Herzegovina	Developing	Medium	[37]
Chalya et al., 2015	436	NA	35.32	Cross-sectional	Tanzania	Developing	Low	[38]
Jaybhaye et al., 2014	220	NA	49.1	Cross-sectional	India	Developing	Medium	[39]
Voide et al., 2012	2.691	NA	9.7	Cross-sectional	Switzerland	Developed	Low	[40]
Shaghaghian et al., 2015	191	43.5	NA	Cross-sectional	Iran	Developing	Medium	[41]

NSI = needle stick injury; NA = not applicable; *N* = sample size.

## Data Availability

All the data are included in the systematic review and meta-analysis. In addition, PRISMA Protocols 2015 checklist is the recommended item to address in a systematic review and meta-analysis.

## Abbreviations

CDC: Centers for Disease Control and Prevention
CMA: Comprehensive meta-analysis
HBV: Hepatitis B virus
HCV: Hepatitis C virus
HCWs: Healthcare workers
HIV: Human immunodeficiency virus
JBI: Joanna Briggs Institute
NSI: Needle stick injury
PRISMA: Preferred Reporting Items for Systematic Review and Meta-Analysis
WHO: World Health Organization.

## Supplementary Materials

Supplementary Materials PRISMA-P (Preferred Reporting Items for Systematic Review and Meta-Analysis) 2015 checklist is one of the recommended items to address in a systematic review.
